# Building a triadic model of technology, motivation, and engagement: a mixed-methods study of AI teaching assistants in design theory education

**DOI:** 10.3389/fpsyg.2025.1624182

**Published:** 2025-07-02

**Authors:** Anlan Wang, Xingting Wu

**Affiliations:** ^1^Academy of Fine Arts, Aba Teachers College, Wenchuan, China; ^2^School of Architecture and Design, Swinburne University of Technology, Melbourne, VIC, Australia

**Keywords:** design education, AI chatbot, mixed methods, task technology fit, self determination theory, student engagement

## Abstract

In design education, it is often more difficult to keep students engaged in theory courses than in hands-on studio classes. Theory courses focus on abstract concepts like design history and principles, which can feel disconnected from practical experience. This study explores how AI-powered teaching assistants can support student engagement in design theory through a mixed-methods approach. Based on Self-Determination Theory (SDT) and Task-Technology Fit (TTF) Theory, we developed a triadic engagement model and tested it with data from 363 undergraduate design students who used a domain-specific AI assistant. Results from Partial Least Squares Structural Equation Modeling (PLS-SEM) and Artificial Neural Networks (ANN) show that communication quality, perceived competence, task-technology fit, and school support are key predictors of engagement. In contrast, individual technology fit and lecturer support have limited effects. Fuzzy-set Qualitative Comparative Analysis (fsQCA) identifies five learner profiles leading to high engagement, showing that different combinations of motivation, support, and technology fit can be effective. Interviews with 10 students identify three themes, further revealing that while the AI assistant is helpful and accessible, it lacks depth in critical thinking, and it demonstrates that students learn to verify AI assistants’ responses and reflect on their learning. This study contributes to education and AI research by showing that chatbots must support both psychological needs and task alignment to foster meaningful engagement. It positions AI not just as an information tool, but as a partner in reflective and autonomous learning.

## 1 Introduction

Design education in China is undergoing rapid transformation (Buchanan, 2004), propelled by the country’s global leadership in both higher education scale and artificial intelligence (AI) development (Yang, 2019). In China’s design education, while Generative AI (GenAI) has been widely introduced into creative design practice, particularly in fields like visual ideation, prototyping, and digital production (Gao et al., 2025). For example, tools like Midjourney support creativity and artistic exploration among design students (Liu et al., 2024), with recent research highlighting a generally high acceptance and openness toward AI-generated content (Shen et al., 2025). However, despite these developments, there remains a notable research gap regarding the application of AI tools in the teaching of theoretical components of design courses. Subjects such as design history, principles, and critical reflection, which involve abstract concepts, often pose challenges in student engagement, compared to the tangible outcomes of studio-based learning. Thus, the potential of GenAI to enhance learning experiences in these theoretical and reflective contexts remains significantly underexplored.

Unlike studio-based learning, which provides immediate, tangible feedback, design theory education demands sustained attention, reflective thinking, and mastery of complex conceptual vocabularies (Chamorro-Koc et al., 2015). Sustaining engagement in such theory-heavy environments is particularly challenging. Traditional hands-on projects offer immediate feedback and visible progress; learning in design theory is slower, more cognitive, and often solitary. Students must navigate not only large volumes of conceptual content but also unfamiliar disciplinary vocabularies. Addressing these challenges requires pedagogical strategies that support reflective, autonomous, and iterative thinking—key elements of design thinking (Schön, 1987; Dorst, 2011). Recent advancements in educational technology, particularly AI-based chatbots, offer new opportunities to support student learning in such contexts. Chatbots can serve as 24/7 virtual assistants, providing immediate answers, personalized feedback, and scaffolded learning support. While the role of AI in education has been extensively studied in STEM and language learning (Zawacki-Richter et al., 2019), recent research has also explored how Chinese university students perceive chatbots in higher education (Tian et al., 2024). However, these studies mostly focus on general fields and lack specificity regarding discipline-based differences. In the Chinese context, students recognize the dual nature of AI chatbots, acknowledging both their potential to support human development and their possible risks and limitations (Zhang et al., 2025). However, how design and art students engage with and evaluate AI chatbots remains underexplored.

**Figure 1:**
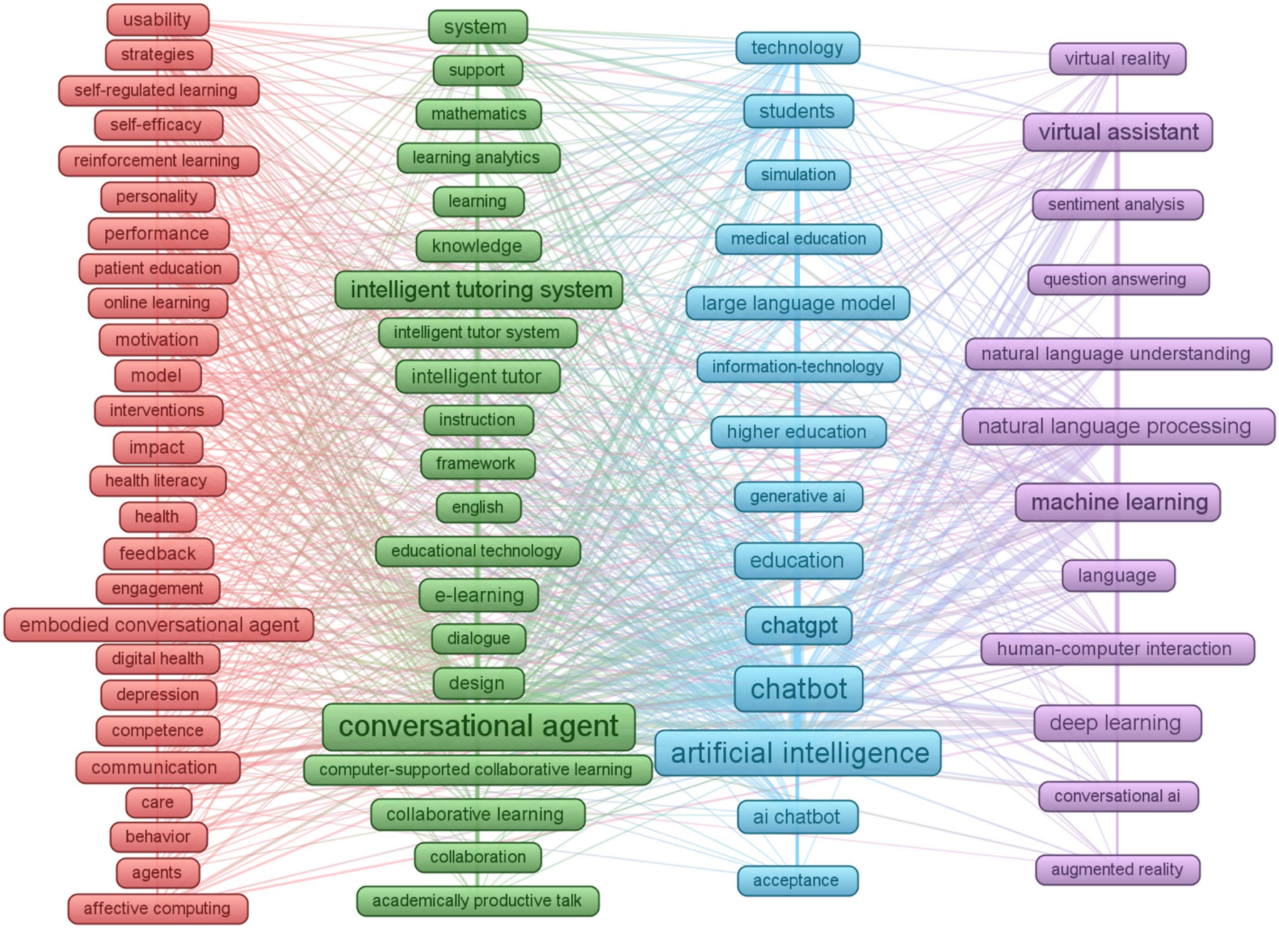
Clustering of AI chatbot research in educational contexts (*n* = 1,276).

To situate this research within the broader field, we conducted a bibliometric analysis using VOSviewer and Web of Science (*n* = 1,276) publications. The results, summarized in Figure 1, reveal four major research clusters:

- A **Human-Centered Design and Application** cluster (red), focusing on usability, learning psychology, pedagogical strategies, and health-related interventions, often involving embodied conversational agents and affective computing.
- An **Intelligent Educational Systems** cluster (green), centered on the design and implementation of intelligent tutoring systems, conversational agents for learning, and computer-supported collaborative learning environments.
- A broader **AI in Education and Technology** cluster (light blue), encompassing the application of artificial intelligence, including large language models like ChatGPT and chatbots, across various educational context s such as higher education, alongside information technology and simulations.
- A Core **AI and Language Technologies** cluster (purple), which underpins many applications and includes fundamental research in machine learning, natural language processing, deep learning, virtual/augmented reality, and human-computer interaction.

While these domains reflect a maturing field, very few studies focus on conceptually driven, practice-linked disciplines such as design and art. Most applications within these clusters prioritize procedural or technical learning and development, leaving a theoretical and emotional engagement gap largely unaddressed. To address these questions, this study develops and explores student engagement with a customized AI-based chatbot assistant—“MinArt AI”—in a Chinese undergraduate design theory course. Grounded on Self-Determination Theory (Deci and Ryan, 1980) and Task-Technology Fit Theory (Goodhue and Thompson, 1995), we propose a triadic model where: Technology attributes (communication quality, task-technology fit, individual-technology fit); Motivational factors (perceived autonomy, perceived competence), and External support (lecturer and school support) collectively influence student engagement.

Employing a mixed-methods design that combines Partial Least Squares Structural Equation Modeling (PLS-SEM), Artificial Neural Networks (ANN), and fuzzy-set Qualitative Comparative Analysis (fsQCA), complemented by qualitative interviews, the study aims to provide a nuanced understanding of how different learner profiles interact with AI teaching assistants. Specifically, the research addresses how psychological, technological, and contextual factors individually and collectively shape student engagement, motivation, and learning experiences.

This study contributes to the broader educational technology literature in several key ways. First, it extends the application of AI-based chatbots into the underexplored domain of design theory education, addressing a major disciplinary gap. Second, it proposes a new triadic framework integrating technology features, motivational dynamics, and engagement outcomes, rather than focusing solely on technology adoption. Third, it applies a hybrid analytical strategy (PLS-SEM, ANN, fsQCA), offering a richer understanding of how different learner profiles interact with AI teaching assistants in design contexts. Finally, it highlights the role of AI not only in supporting information retrieval but also in fostering reflective, self-regulated learning practices essential for design education.

Specifically, this study seeks to answer the following overarching research question:

*How do psychological, technological, and contextual factors shape student engagement with AI teaching assistants in design theory education?*

We investigate several sub-questions:

- **RQ1:** How do students’ perceptions of autonomy and competence influence their engagement?
- **RQ2:** How does the communication quality of AI teaching assistants contribute to student engagement and motivation?
- **RQ3:** How do perceived task-technology fit and individual-technology fit influence student motivation and engagement?
- **RQ4:** How do contextual supports (lecturer and institutional support) influence student engagement?
- **RQ5:** What combinations of these factors lead to high levels of engagement?
- **RQ6:** How do students describe their experiences using AI teaching assistants in design theory courses?

By exploring these questions, this research seeks to contribute a more nuanced understanding of how AI tools can be meaningfully integrated into conceptually complex, creativity-driven educational settings.

## 2 Theoretical background and hypothesis development

### 2.1 Supporting student motivation through self-determination theory

Self-Determination Theory (SDT), developed by Deci and Ryan (1980) and Ryan et al. (2022), offers a comprehensive psychological framework to understand human motivation. At its core, SDT posits that individuals are driven by the innate psychological needs for autonomy (the desire to self-regulate one’s actions), competence (the need to feel capable and effective), and relatedness (the sense of connection with others). When these needs are satisfied, individuals are more likely to develop intrinsic motivation, resulting in enhanced engagement, well-being, and personal growth.

In educational contexts, SDT has been extensively applied to understand how learning environments can support students’ self-regulated learning, motivation, and academic outcomes (Wang Y. et al., 2024; Wu and Chiu, 2025). The theory emphasizes that supportive learning conditions that foster autonomy and competence are crucial for sustaining students’ participation and deep learning.

With the increasing integration of digital technologies into education, SDT has found renewed relevance. In digital learning environments, satisfying the need for autonomy and competence is especially vital, as students often engage with learning tools and content in more self-directed ways. Studies have demonstrated that digitally mediated learning systems—including AI-enhanced tools—can effectively support these needs and improve engagement (Chiu, 2022).

In the context of AI-powered learning assistants such as chatbots, SDT offers a powerful lens to examine how these tools influence learner motivation. For example, an AI chatbot that enables learners to control their pace of study (autonomy) (Alamri et al., 2020), offers timely and constructive feedback (competence), and facilitates meaningful learning interactions (relatedness) may foster deeper engagement (Chiu et al., 2024). Thus, SDT provides a theoretically grounded foundation for examining how students’ perceptions of AI-enabled educational technologies shape their emotional and cognitive involvement in learning (Li et al., 2024).

### 2.2 Aligning technology and learning tasks: task-technology fit theory

Task-Technology Fit (TTF) theory, originally proposed by Goodhue and Thompson (1995), addresses how well a given technology aligns with the specific tasks users are trying to accomplish. According to the theory, the effectiveness of technology use is maximized when the technological features appropriately match the task requirements (Suhail et al., 2024). In other words, a high degree of task-technology fit enhances the likelihood that individuals will perceive the technology as useful and adopt it more readily, ultimately improving performance outcomes (Sharma et al., 2024).

The TTF model includes multiple constructs—task characteristics, technology characteristics, utilization, and performance impact—all of which converge on the central idea that technology should serve the actual needs of its users (Saifi et al., 2025; Wang C. et al., 2024). For example, if students perceive that an AI teaching assistant helps them complete coursework, access relevant information, or enhance comprehension efficiently, the fit between the tool and the academic task is considered high.

Therefore, TTF provides an important complementary framework to SDT in this study. While SDT focuses on internal motivational mechanisms, TTF emphasizes the external functional alignment between technology and educational needs. Together, these frameworks enable a comprehensive exploration of how AI teaching assistants influence student engagement by simultaneously addressing what learners feel (motivation) and how learners act (task performance fit).

### 2.3 Hypothesis development

#### 2.3.1 Student engagement

Student engagement has evolved from a narrow behavioral concept to a comprehensive, multidimensional construct encompassing emotional, cognitive, behavioral, and even social and agentic dimensions. Early models, such as Natriello’s (1984) participation-based view and Finn’s (1989) participation-identification model, primarily focused on observable behaviors and affective affiliation with school. Later, frameworks like the Self-System Model of Motivational Development (Skinner et al., 2009), grounded in Self-Determination Theory, emphasized the psychological needs of autonomy, competence, and relatedness as drivers of engagement. The concept of “flow” (Csikszentmihalyi and Csikzentmihaly, 1990) introduced engagement as a dynamic and intrinsically rewarding state of deep concentration and enjoyment, while schoolwork engagement perspectives highlighted enduring motivational states such as vigor, dedication, and absorption (Salmela-Aro and Upadaya, 2012). Later refinements include Fredricks et al.’s (2004) tripartite framework (behavioral, emotional, and cognitive engagement) and its subsequent extensions to include agentic (Reeve and Tseng, 2011) and social engagement (Fredricks et al., 2016). These developments underscore engagement as both context-sensitive and student-driven, shaped by internal motivation and external educational environments.

Recent literature has emphasized the multifaceted nature of engagement, proposing distinct yet interconnected dimensions: behavioral (active participation and effort), cognitive (psychological investment and strategic learning), and emotional (feelings of connection and interest) (Fredricks et al., 2004; Wong et al., 2024). Studies consistently show behavioral engagement, manifested through persistent effort and active participation, as having the strongest direct relationship with academic achievement (Lam et al., 2014; Skinner et al., 2009). Cognitive engagement, involving deeper processing and self-regulated learning strategies, also significantly predicts academic performance (Reeve and Tseng, 2011). Affective engagement, representing emotional investment, though less directly correlated, is critical for sustaining long-term motivation and academic persistence (Fredricks and McColskey, 2012).

Engagement is widely recognized as a key predictor of academic success and well-being. Student engagement has been described as “the holy grail of learning.” Student engagement is widely recognized as a critical construct in educational psychology due to its significant associations with a range of desirable educational outcomes, including academic achievement and subjective well-being (Wong et al., 2024). Consequently, it is often investigated as a key dependent variable, reflecting students’ success and adaptation within the educational system (Hoi and Le Hang, 2021; Maricuțoiu and Sulea, 2019; Merkle et al., 2022).

In digital learning contexts, especially with the rise of AI, there is growing evidence of technology’s potential to enhance student engagement. Studies indicate that digital interventions can stimulate cognitive and emotional engagement (Guo et al., 2024). Specifically, AI-powered teaching assistants and chatbots have shown promise in promoting active interaction, immediate feedback, and personalized learning experiences, thus positively impacting behavioral and cognitive engagement dimensions (Enriquez et al., 2023; Hidayat-ur-Rehman, 2024b). For example, AI chatbots offer continuous, responsive support tailored to individual student queries, fostering deeper cognitive processing and sustained learning motivation (Nguyen et al., 2024). Furthermore, GenAI significantly propels the personalized learning experience by facilitating real-time interactions with each learner and tailoring learning materials and feedback according to individual student needs (Xie et al., 2019; Zheng et al., 2021). This capacity for dynamic personalization, coupled with the anytime, anywhere accessibility of GenAI tools, supports a variety of learning styles and schedules, thereby increasing student engagement and enriching the overall learning experience. In addition, the immediate and tailored feedback provided by AI, for instance, can shape students’ cognitive engagement experiences by clarifying misconceptions quickly, while its continuous availability may foster a more positive affective experience by reducing learning anxiety. Such AI-facilitated engagement experiences, where students feel more competent, autonomous, and emotionally connected to the learning process (Ait Baha et al., 2024), are posited to serve as crucial mediating pathways to enhanced educational outcomes.

#### 2.3.2 The influence of SDT on student engagement

SDT posits that human motivation and engagement are largely shaped by the satisfaction of three basic psychological needs: autonomy, competence, and relatedness (Deci and Ryan, 1980; Ryan et al., 2022). When these needs are supported in educational settings, students are more likely to exhibit intrinsic motivation, sustained effort, and higher levels of academic engagement.

Among these needs, autonomy—the sense of having control and choice in one’s learning—has been shown to play a particularly central role in digital learning environments (Chiu, 2022). Autonomy-supportive settings encourage students to make their own decisions about how and when to learn, which leads to increased persistence, engagement, and satisfaction (Hidayat-Ur-Rehman, 2024a). As Connell (1990) explains, autonomy reflects the perception that one’s actions align with personal goals and values, fostering a sense of ownership in learning.

With the rise of AI-powered tools such as chatbots, learners are now able to access support anytime, anywhere. This flexibility enhances students’ perceived autonomy by allowing them to freely explore questions, choose learning paths, and control the pace of their study. Unlike human teachers, AI assistants do not fatigue and can respond instantly to repeated or complex queries (Annamalai et al., 2023). This 24/7 availability creates a self-directed learning environment where students feel empowered and more engaged in their academic experience (Lai et al., 2023; Segbenya et al., 2023).

In addition to autonomy, the need for competence—feeling effective and capable in learning—also contributes significantly to student engagement (Losier and Vallerand, 1994). When students believe they can succeed in using digital tools and AI systems, they are more likely to remain motivated and actively involved (Li et al., 2024). AI chatbots, by offering immediate feedback and personalized explanations, can help students build confidence and overcome learning difficulties, reinforcing their sense of competence and encouraging deeper participation. Prior studies have confirmed that when classroom environments support students’ autonomy and competence, learners are better prepared to engage and perform (Yang et al., 2022).

Based on the previous research, we propose the following hypotheses:

*H1:* Students’ perceived autonomy positively influences their engagement.

*H2:* Students’ perceived competence positively influences their engagement.

#### 2.3.3 Chatbots’ communication quality

Communication quality plays a pivotal role in shaping users’ trust, satisfaction, and engagement with digital agents. Prior research in marketing and service contexts has shown that when service agents—whether human or AI-based—deliver accurate, relevant, and reliable information, users are more likely to feel satisfied, reduce uncertainty, and form lasting connections (Barry and Crant, 2000; Chung et al., 2020; Mohr and Sohi, 1995). In digital environments, chatbots are increasingly expected to replicate these standards of quality communication, including in educational contexts.

Communication quality has been commonly defined by three core attributes: accuracy, credibility, and competence (Chung et al., 2020; Edwards et al., 2014). Accurate communication ensures the clarity and correctness of information (Barry and Crant, 2000); credibility fosters trust and emotional acceptance (O’Keefe, 2006); and communication competence refers to the chatbot’s ability to respond appropriately, efficiently, and contextually (Spitzberg, 2006).

In educational settings, particularly within AI-powered learning environments, communication quality has been found to significantly influence student engagement. For instance, chatbots have been used to support students in writing (Shafiee Rad, 2025), vocabulary learning (Song et al., 2023), and reading activities (Liu et al., 2022), all showing positive impacts on participation and learning involvement. Wang and Xue (2024) further demonstrated that students were more engaged in academic tasks when supported by AI-driven chat interfaces that provided timely, competent, and trustworthy feedback.

In design theory education, where abstract concepts and complex historical knowledge are involved, communication quality becomes even more critical. Students not only need factual correctness, but also contextual interpretation, conceptual clarity, and tailored feedback—dimensions that high-quality AI communication can potentially fulfill.

Based on the previous research, we propose the following hypotheses:

*H3:* The communication quality of AI chatbots positively influences student engagement.

#### 2.3.4 Task-technology fit and individual-technology fit

TTF refers to how well a technology supports the tasks that users need to perform (Goodhue and Thompson, 1995). In the context of education, TTF describes whether a tool, such as an AI teaching assistant, can effectively help students complete learning activities, such as understanding concepts, conducting research, or writing reports (Saifi et al., 2025). When students perceive that the chatbot is useful for their learning tasks, they are more likely to engage actively with the course (Al-Emran et al., 2025; Lousã and Lousã, 2023).

Individual-Technology Fit (ITF) focuses on the match between the technology and the learner’s personal abilities and preferences (Goodhue and Thompson, 1995). It considers whether students feel confident and motivated to use the tool on their own (Wu and Chen, 2017). When students believe the AI chatbot fits their personal learning style, such as self-directed learning or exploratory questioning, they are more likely to use it frequently and meaningfully.

Based on this reasoning, the following hypotheses are proposed:

*H4:* The task–technology fit (TTF) of AI teaching assistants positively influences student engagement.

*H5:* The individual–technology fit (ITF) of AI teaching assistants positively influences student engagement.

#### 2.3.5 The mediation effect of TTF and SDT in AI chatbots facilitates student engagement

In educational contexts, perceived autonomy and perceived competence are key motivational factors that influence how students interact with learning technologies (Collie and Martin, 2024).

AI chatbots, when well-designed, offer timely, personalized feedback that helps learners feel more in control of their study process. This sense of control aligns with the concept of autonomy, enabling students to make their own learning decisions (Chiu et al., 2023). In fact, prior research shows that when learners feel autonomous and capable, their motivation, persistence, and satisfaction increase (Soliman et al., 2024).

Therefore, the following hypotheses are proposed:

*H6:* The communication quality of AI teaching assistants positively influences students’ perceived autonomy.

*H7:* The communication quality of AI teaching assistants positively influences students’ perceived competence.

At the same time, ITF refers to how well a student’s personal characteristics and preferences align with a technology. When students feel that the chatbot suits their learning style and they can easily interact with it, they are more likely to feel confident and in control of their learning. Research has shown that ITF is positively related to ease of use, perceived usefulness, and autonomous motivation (Racero et al., 2020; Rezvani et al., 2017).

Therefore, the following hypotheses are proposed:

*H8:* The individual–technology fit (ITF) of AI teaching assistants positively influences students’ perceived autonomy.

*H9:* The individual–technology fit (ITF) of AI teaching assistants positively influences students’ perceived competence.

Similarly, TTF assesses whether the technology meets the requirements of learning tasks. If students find the chatbot helpful for understanding theories, answering questions, and completing assignments, they are more likely to feel that the tool supports their goals. TTF has been found to enhance both student engagement and learning outcomes in digital environments (B. Wu and Chen, 2017). As students achieve learning goals with the help of AI tools, their sense of competence and autonomy may increase. Therefore:

*H10:* The task–technology fit (TTF) of AI teaching assistants positively influences students’ perceived autonomy.

*H11:* The task–technology fit (TTF) of AI teaching assistants positively influences students’ perceived competence.

#### 2.3.6 External support from the school and lecturers

Institutional and teacher support play a critical role in shaping how students engage with AI-powered tools in educational settings (Molefi et al., 2024). Previous research shows that schools are more likely to adopt AI technologies when they believe such solutions can enhance teaching effectiveness and improve student outcomes (Fokides, 2017). Studies reveal that school resources and support play an important role in facilitating the effective integration of AI-driven technologies into classrooms (Druga et al., 2022). Tao et al. (2022) found through meta-analysis that perceived teacher support is strongly associated with student academic success, particularly emotional support. In AI-enhanced learning environments, the role of the teacher becomes even more important. Teachers guide students in using AI tools effectively, designing learning tasks, and providing emotional and instructional support (Chiu et al., 2024; Pitzer and Skinner, 2017). However, potential risks also exist, such as students becoming overly dependent on chatbots, concerns around academic integrity, plagiarism, or encountering biased or incorrect content (Gammoh, 2025; Williams, 2024). To manage these risks, institutions must establish clear guidelines and ethical frameworks to support the responsible use of AI in learning.

Based on these findings, this study proposes the following hypotheses:

*H12:* Perceived school support for the use of AI teaching assistants positively influences student engagement in design education.

*H13:* Perceived lecturer support for the use of AI teaching assistants positively influences student engagement in design education.

### 2.4 Integrating motivation, technology fit, and external support: a triadic engagement model

Bringing together Self-Determination Theory (SDT), Task-Technology Fit (TTF), and the role of External Support, this study constructs a Triadic Engagement Model to explain how AI teaching assistants facilitate student engagement in design theory education.

Specifically, the model proposes three key pathways:

- **Motivational Pathway**: Students’ perceived autonomy and competence, influenced by chatbot communication quality and technology fit, drive intrinsic engagement.
- **Cognitive-Functional Pathway:** Task-Technology Fit and Individual-Technology Fit ensure that the AI assistant supports students’ learning needs and personal learning styles.
- **Contextual Support Pathway:** External encouragement and infrastructure provided by institutions and lecturers further amplify the use and integration of AI tools.

**Figure 2:**
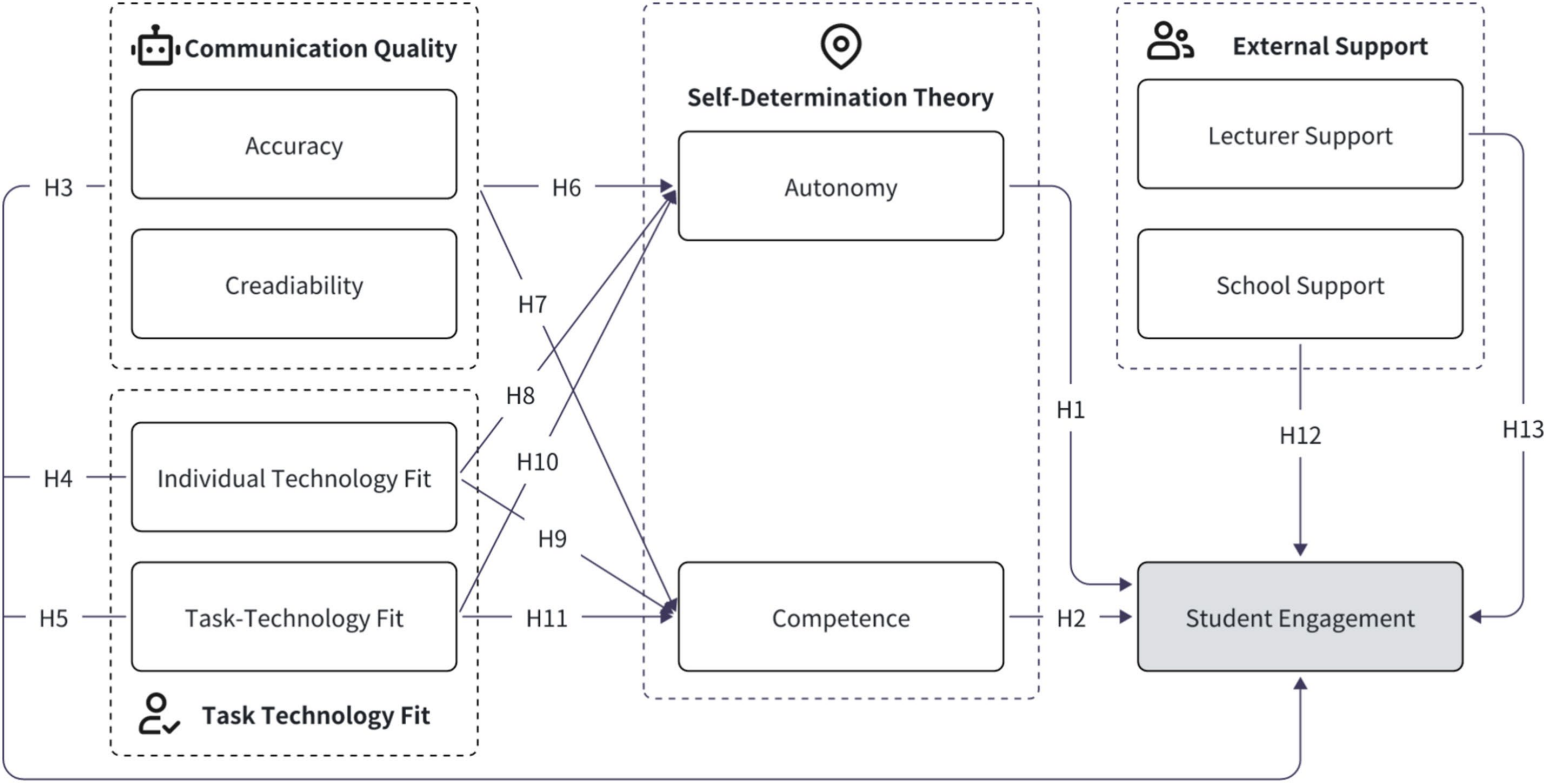
Research model.

This integrative model moves beyond traditional technology adoption frameworks by highlighting the interplay between internal psychological needs, task-technology alignment, and social-structural support in shaping engagement with AI-powered tools in design education. Through this approach, the study addresses a critical gap in the AI-in-education literature by focusing not just on adoption or functionality, but also on the emotional, motivational, and technology dimensions of student experience. Based on the proposed Triadic Engagement Model, this study addresses one overarching question: *How do psychological, technological, and contextual factors shape student engagement with AI teaching assistants in design theory education?* through six specific sub-questions, each linked to particular theoretical variables and hypotheses. Table 1 presents the overview of research questions, theoretical basis, and hypotheses. Figure 2 shows the proposed research model and hypotheses.

**Table 1:** Overview of research questions, theoretical basis, and hypotheses.

Research question	Theoretical component	Independent variable(s)	Dependent variable(s)	Linked hypothesis/method
**RQ1**: How do students’ perceptions of autonomy and competence influence their engagement?	SDT	PA, PC	SE	H1, H2
**RQ2**: How does the communication quality of AI teaching assistants contribute to student engagement and motivation?	Technology to Engagement and Motivation	CQ	SE, PA, PC	H3, H6, H7
**RQ3**: How do perceived task-technology fit and individual-technology fit influence student motivation and engagement?	TTF	TTF, ITF	SE, PA, PC	H4, H5, H8–H11
**RQ4**: How do contextual supports (lecturer and institutional support) influence student engagement?	Contextual Support	LS, SS	SE	H12, H13
**RQ5**: What combinations of these factors lead to high levels of engagement?	Configurational	All above variables jointly	SE	fsQCA Analysis
**RQ6**: How do students describe their experiences using AI teaching assistants in design theory courses?	Qualitative Inquiry	Not applicable	Student experiences, perception, and reflection	Semi-structured Interviews and Thematic Analysis

## 3 Research methodology

To comprehensively address the research questions in this study, a mixed-methods design was employed. This approach integrates quantitative techniques—Partial Least Squares Structural Equation Modeling (PLS-SEM), Artificial Neural Networks (ANN), and fuzzy-set Qualitative Comparative Analysis (fsQCA)—with qualitative thematic analysis of interview data. This combination allows for both hypothesis testing and a rich contextual understanding of students’ engagement with AI teaching assistants in design theory education.

The rationale for adopting this mixed-methods design lies in its capacity to offer a more comprehensive and nuanced understanding of the complex phenomenon of student engagement with AI teaching assistants than either a purely quantitative or qualitative approach could yield (Creswell, 1999). The quantitative phase, encompassing PLS-SEM, ANN, and fsQCA, was designed to identify significant predictors of engagement, model their interrelationships, and uncover common configurations of factors leading to high engagement, thereby addressing research questions RQ1 through RQ5. Complementing this, the qualitative phase, through semi-structured interviews (RQ6), aimed to delve into students’ subjective experiences, providing rich, contextual details that help explain the quantitative findings and explore the underlying reasons for observed patterns. This sequential explanatory design allows for the qualitative data to elaborate upon and add depth to the statistical results, facilitating a more robust interpretation and offering actionable insights for integrating an AI teaching assistant into design theory education.

### 3.1 AI teaching assistant development and survey instrument

The AI teaching assistant used in this study, named “MinArt AI,” was developed by the first author, a senior university lecturer with decades of experience teaching design theory courses such as History of Chinese and Foreign Design and History of Chinese Craft and Decorative Arts. The name “MinArt” reflects both geographical and cultural significance: “Min” refers to the Minjiang River, a major river in the Aba Prefecture where the university is located, often seen as a cultural symbol or “mother river” of the region. “Art” signifies the chatbot’s foundation in the arts. The long-term vision for MinArt AI is to evolve into a comprehensive educational agent that spans both fine arts and design domains, offering students extensive support across disciplines. Based on the existing commercial AI large language model ERNIE Bot, a commercial AI large language model, the author fine-tuned a domain-specific AI chatbot assistant using a curated corpus of design theory resources. Appendix A presents the technical documentation.

**Figure 3:**
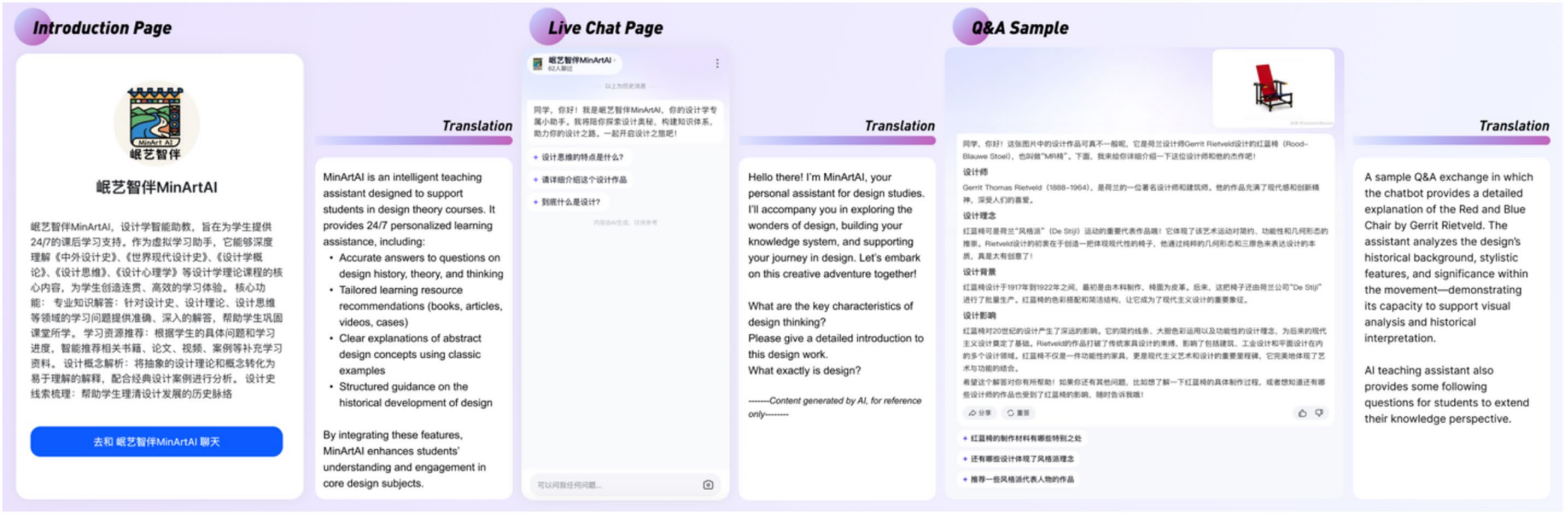
AI teaching assistant user interfaces.

“MinArt AI” was deployed through a course web portal and made available to students 24/7. Its core functionalities included answering lecture-related questions, providing feedback on design concepts, and recommending supplementary examples. This direct integration positioned the AI assistant as an embedded element of the learning process rather than an ancillary tool. Figure 3 shows the homepage of the AI teaching assistant and a sample interaction in which students asked questions about the Red and Blue Chair. The AI teaching assistant is publicly accessible at https://mbd.baidu.com/ma/s/0gjlz9Z7.

The questionnaire items used in this study were developed based on prior research and measured using a five-point Likert scale. To ensure content validity, the survey underwent both a pre-test and a pilot test before the actual data collection. Since the present study was carried out in China, the questionnaire was developed using Back-translation (Brislin, 1986). Two bilingual experts from the College of Foreign Languages independently translated the survey from English to Chinese and then back into English to ensure accuracy in meaning and terminology. Appendix B presents the questionnaires.

### 3.2 Sampling and data gathering

This study received ethical approval from the University Research Ethics Committee. To ensure the privacy of respondents, several key issues were addressed before they completed the questionnaire. Participants were informed that all collected data would be used solely for academic purposes, participation was entirely voluntary, and their responses would remain anonymous. Informed consent was obtained once participants agreed to these terms.

The participants were undergraduate students majoring in various art and design disciplines, including visual communication, fine arts, and environmental design. They were enrolled across multiple year levels and degree programs and had all taken one or more compulsory design theory courses, such as History of Chinese and Foreign Design and Design Theory. All participants had prior experience using the “MinArt AI” teaching assistant during their coursework. Data were collected through a combination of online distribution via course communication platforms and offline dissemination using QR codes during class sessions. In total, 400 questionnaires were distributed.

After data collection, duplicate and ineligible responses were screened and removed. The following invalid responses were excluded. For online questionnaires: (1) Responses completed in less than 1 min; (2) Responses that failed the attention check at the end of the questionnaire. Following the exclusion of responses with absent values and outliers, 363 questionnaires were used for subsequent analysis (Ngoc Su et al., 2023).

According to the suggestion of Soper (2020), we determined the prior sample size for the structural equation model. This involves using an online power analysis application with the URL: https://www.danielsoper.com/statcalc/calculator.aspx?id=89. The inputs considered by our research model, which include the number of observed variables (43 items measuring constructs in our study) and latent variables (a total of 9 variables), expected effect size (medium effect is 0.25), expected probability (95% significance level), and statistical power level (80%). The online power analysis application determined that the minimum sample size required to detect the specified effect is 281 based on the structural complexity of the model. The sample size used in this study (*N* = 363) is larger than the recommended amount, indicating an adequate sample size. Besides, to gain deeper insights into students’ real-world experiences with AI teaching assistants, we conducted semi-structured interviews with 10 undergraduate students enrolled in the History of Chinese and Foreign Design course.

### 3.3 Quantitative phase (RQ1–RQ5)

To explore RQ1–RQ4, PLS-SEM was conducted using SmartPLS 4.0 to examine how different technological, motivational, and contextual factors influence students’ engagement with the AI teaching assistant in design theory courses. PLS-SEM was chosen due to its suitability for handling complex models with multiple latent constructs and its robustness in small to medium sample sizes, which is critical for understanding the complexity of the student’s adoption of the technology situation (Al-Emran et al., 2025; Hair et al., 2017). This technique enabled us to test direct and mediating effects between constructs.

To complement the linear findings from PLS-SEM and capture possible nonlinear relationships, we applied ANN analysis using SPSS 27. ANN helps identify the relative importance of significant predictors and enhances model prediction accuracy (Kalinić et al., 2021; Leong et al., 2019). This is important in an educational setting because many interacting factors influence student behaviors and attitudes toward technology (Al-Emran et al., 2025). A two-layer feedforward network with 10-fold cross-validation was constructed to avoid overfitting and ensure the generalizability of results.

To explore RQ5, we adopted fuzzy-set Qualitative Comparative Analysis (fsQCA) to uncover multiple conjunctural paths that lead to high student engagement (Abbasi et al., 2022). This method is particularly suitable for identifying causal complexity and understanding how combinations of conditions may be sufficient for the outcome (Ragin, 2009).

### 3.4 Qualitative phase: semi-structured interviews (RQ6)

To gain deeper insight into students’ experiences using AI teaching assistants in design theory education, the first author conducted semi-structured interviews with 10 undergraduate students who had interacted deeply with the AI teaching assistant throughout the semester. The participants were purposefully selected from multiple class cohorts to ensure diversity in academic background and AI teaching assistant usage frequency. The interviews were guided by open-ended prompts, such as: “In what ways has the AI teaching assistant helped you understand design theory content?,” “What limitations have you encountered?,” and “How does it compare to traditional teaching methods?” All interviews were conducted in a quiet setting, audio-recorded with participant consent, and transcribed verbatim by the first author to maintain data accuracy.

The qualitative data were then analyzed following Braun and Clarke’s (2019) six-step reflexive thematic analysis. Both authors independently read and coded all transcripts, identifying key phrases and patterns. Through iterative discussion, the codes were refined, grouped, and developed into broader themes. Any discrepancies in interpretation were resolved through dialogue until consensus was reached, ensuring analytical rigor and intersubjective reliability.

## 4 Data analysis and results

### 4.1 Common method variance

Considering the CMV that the questionnaire survey method could have potentially caused in this study, the Harman single-factor test was applied as a post-hoc statistical control (Al-Emran et al., 2025; Howard et al., 2024). The subsequent principal component factor analysis indicated that the first factor accounted for only 34.601% of the variance, which is well below the 50% threshold (Podsakoff et al., 2003). Furthermore, the variance inflation factors (VIF) of all latent constructs were calculated to test the risk of multi-collinearity, and all of these were inferior to the vigilance value of 5 (Hair et al., 2017). Therefore, CMV is not considered a significant issue in this study.

### 4.2 Measurement model assessment

To ensure the quality, internal consistency, and reliability of the constructs, this study conducted validity and reliability tests following the recommendations of Hair et al. (2019). Cronbach’s alpha (*α*) and composite reliability (CR) were used to assess internal consistency and reliability. The results showed that both *α* and CR values exceeded the 0.70 threshold, indicating strong internal consistency and construct reliability. Convergent validity was supported by Average Variance Extracted (AVE), as all AVE values were above 0.50, confirming an adequate level of convergent validity (Chen et al., 2024). The detailed results, including α, CR, AVE, and factor loadings, are presented in Table 2.

**Table 2:** Reliability and convergent validity analysis.

Construct	Item	M	SD	FL	*α*	CR	AVE
Communication quality					0.874	0.883	0.529
Accuracy	AC1	4.039	0.805	0.747			
	AC2	3.917	0.809	0.780			
	AC3	4.080	0.765	0.716			
	AC4	4.000	0.768	0.775			
Credibility	CRE1	4.069	0.802	0.687			
	CRE2	4.066	0.873	0.756			
	CRE3	4.116	0.770	0.679			
	CRE4	4.190	0.810	0.671			
Individual Technology Fit	ITF1	4.028	0.809	0.825	0.830	0.843	0.662
	ITF2	4.052	0.823	0.749			
	ITF3	3.975	0.870	0.823			
	ITF4	4.033	0.905	0.854			
Technology Fit	TTF1	4.072	0.720	0.798	0.815	0.817	0.576
	TTF2	3.964	0.765	0.700			
	TTF3	4.039	0.756	0.715			
	TTF4	4.110	0.703	0.780			
	TTF5	4.113	0.689	0.796			
Perceived Autonomy	PA1	3.981	0.895	0.861	0.857	0.870	0.636
	PA2_R	3.556	1.113	0.741			
	PA3	3.912	0.808	0.790			
	PA4	3.997	0.851	0.841			
	PA5_R	3.565	1.072	0.747			
Perceived Competence	PC1_R	3.584	1.055	0.778	0.821	0.836	0.585
	PC2	3.683	0.828	0.801			
	PC3	4.025	0.818	0.800			
	PC4	3.788	0.857	0.805			
	PC5_R	3.372	1.148	0.623			
Lecturer Support	LS1	3.570	0.914	0.743	0.816	0.818	0.578
	LS2	3.942	0.782	0.816			
	LS3	4.099	0.701	0.677			
	LS4	3.868	0.799	0.801			
	LS5	3.945	0.780	0.757			
School Support	SS1	3.873	0.807	0.874	0.852	0.856	0.693
	SS2	3.975	0.762	0.832			
	SS3	3.680	0.926	0.815			
	SS4	3.992	0.751	0.808			
Student Engagement	SE1	4.022	0.676	0.686	0.857	0.857	0.538
	SE2	4.083	0.760	0.767			
	SE3	3.970	0.762	0.738			
	SE4	4.058	0.734	0.749			
	SE5	4.033	0.699	0.730			
	SE6	4.107	0.690	0.708			
	SE7	3.887	0.797	0.755			

M, mean; SD, standard deviation; FL, factor loading; *α*, Cronbach’s alpha; CR, composite reliability; AVE, average variance extracted.

Discriminant validity was evaluated using two methods. First, the Heterotrait-Monotrait ratio (HTMT) results in Table 3 were all below the 0.90 cutoff (Henseler et al., 2015). Second, the Fornell-Larcker criterion, presented in Table 4, showed that the square root of each construct’s AVE was greater than its correlations with other constructs (Fornell and Larcker, 1981). Together, these results indicate that the constructs used in this study demonstrated adequate reliability, convergent validity, and discriminant validity.

**Table 3:** HTMT ratios evaluation.

Constructs	CQ	ITF	LS	PA	PC	SS	SE	TTF
CQ								
ITF	0.552							
LS	0.582	0.574						
PA	0.549	0.437	0.401					
PC	0.572	0.502	0.483	0.770				
SS	0.601	0.529	0.880	0.436	0.539			
SE	0.708	0.585	0.693	0.663	0.735	0.764		
TTF	0.613	0.569	0.682	0.570	0.579	0.742	0.782	

PA, Perceived autonomy; PC, Perceived competence; CQ, Communication quality; ITF, Individual technology fit; TTF, Task-technology fit; SS, School support; LS, Lecturer support; SE, Student engagement.

**Table 4:** Fornell-Larcker criterion.

Constructs	CQ	ITF	LS	PA	PC	SS	SE	TTF
CQ	**0.728**							
ITF	0.478	**0.814**						
LS	0.493	0.473	**0.760**					
PA	0.503	0.388	0.350	**0.797**				
PC	0.508	0.429	0.408	0.642	**0.765**			
SS	0.530	0.447	0.731	0.391	0.468	**0.832**		
SE	0.626	0.499	0.583	0.573	0.621	0.657	**0.734**	
TTF	0.529	0.465	0.554	0.484	0.482	0.618	0.655	0.759

Bold-faced diagonal elements are the square roots of AVEs. The off-diagonal elements are the correlations between constructs. PA, Perceived autonomy; PC, Perceived competence; CQ, Communication quality; ITF, Individual technology fit; TTF, Task-technology fit; SS, School support; LS, Lecturer support; SE, Student engagement.

### 4.3 Structural model assessment

In the second phase, the structural model was assessed following the recommendations of Hair et al. (2019). The Bootstrap method with 5,000 bootstrap samples was employed to conduct significance testing within a 95% confidence interval. The analysis focused on standardized path coefficients (*β*), *p*-values, and the coefficient of determination (*R*^2^) to evaluate the model’s explanatory power (Latif et al., 2024).

As shown in Table 5, most hypotheses were supported. Specifically, perceived autonomy (PA), perceived competence (PC), communication quality (CQ), task-technology fit (TTF), and school support (SS) all had significant positive effects on student engagement (SE), confirming H1, H2, H3, H5, and H12, respectively. However, H4 (ITF → SE) and H13 (lecturer support → SE) were not supported due to non-significant p-values. In addition, communication quality significantly influenced both perceived autonomy and competence (H6, H7), while both individual-technology fit (ITF) and task-technology fit (TTF) significantly influenced PA and PC (H8–H11). According to Hair et al. (2019), *R*^2^ values of 0.25, 0.50, and 0.75 indicate weak, moderate, and substantial explanatory power, respectively. The *R*^2^ values were 0.652 for SE, 0.329 for PA, and 0.437 for PC, indicating moderate to substantial explanatory power.

**Table 5:** Hypothesis testing results.

Hypothesis	Path	ß	*p* Value	Remark
H1	PA → SE	0.127	0.016**	Yes
H2	PC → SE	0.196	0.000***	Yes
H3	CQ → SE	0.177	0.001**	Yes
H4	ITF → SE	0.054	0.292^n/s^	No
H5	TTF → SE	0.207	0.000***	Yes
H6	CQ → PA	0.305	0.000***	Yes
H7	CQ → PC	0.295	0.000***	Yes
H8	ITF → PA	0.116	0.047*	Yes
H9	ITF → PC	0.174	0.003**	Yes
H10	TTF → PA	0.269	0.000***	Yes
H11	TTF → PC	0.245	0.000***	Yes
H12	SS → SE	0.216	0.004**	Yes
H13	LS → SE	0.073	0.197 ^n/s^	No

PA, Perceived autonomy; PC, Perceived competence; CQ, Communication quality; ITF, Individual technology fit; TTF, Task-technology fit; SS, School support; LS, Lecturer support; SE, Student engagement.

Mediation analysis (see Table 6) further revealed several significant indirect effects. PC partially mediated the relationships between CQ, ITF, and TTF with SE, while PA also showed some mediation effects, although weaker. Specifically, the path CQ → PC → SE had the strongest indirect effect (*β* = 0.058, *p* = 0.010), confirming the importance of perceived competence in facilitating AI teaching assistant-driven engagement.

**Table 6:** Mediating effect test.

Indirect effects	Path	ß	*p* Value
Partial mediation effect	CQ - > PC - > SE	0.058	0.010**
Partial mediation effect	CQ - > PA - > SE	0.039	0.043*
Partial mediation effect	ITF - > PC - > SE	0.034	0.026*
No mediation effect	ITF - > PA - > SE	0.015	0.167 ^n/s^
Partial mediation effect	TTF - > PC - > SE	0.048	0.006**
Partial mediation effect	TTF - > PA - > SE	0.034	0.035*

PA, Perceived autonomy; PC, Perceived competence; CQ, Communication quality; ITF, Individual technology fit; TTF, Task-technology fit; SE, Student engagement.

**Figure 4:**
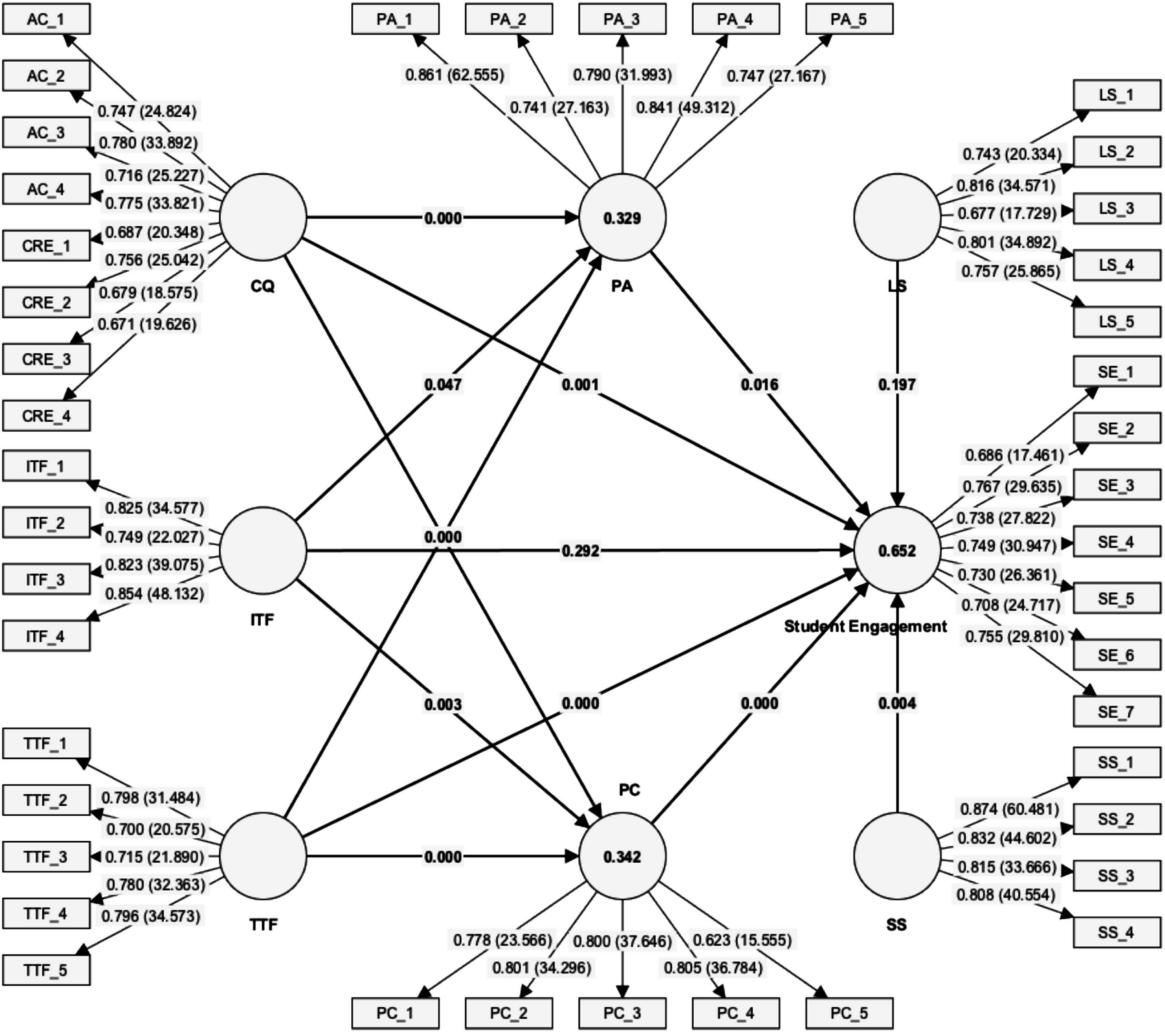
Structural model results. Note: *p < 0.05; **p < 0.01; ***p < 0.001.

Figure 4 illustrates the final structural model with path coefficients and *R*^2^ values.

### 4.4 ANN analysis

**Figure 5:**
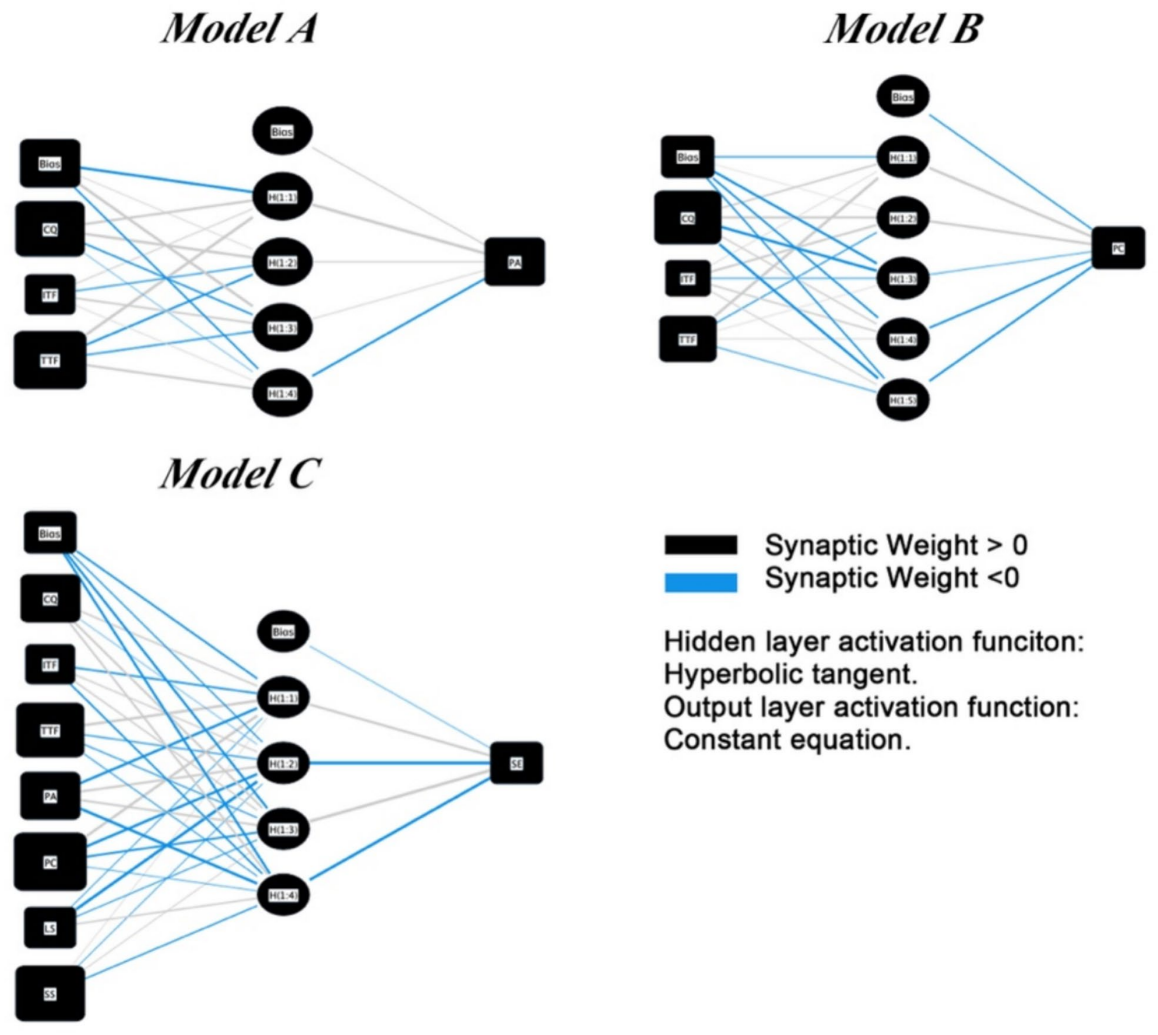
Artificial neural network diagram.

To complement the linear structural model and further explore complex nonlinear relationships, this study developed three ANN models to predict Perceived Autonomy (PA), Perceived Competence (PC), and Student Engagement (SE). As shown in Figure 5, all models used Communication Quality (CQ), Individual Technology Fit (ITF), and Task-Technology Fit (TTF) as key input variables, with additional variables (PA, PC, LS, SS) added in the SE model.

Model performance was evaluated using Root Mean Square Error (RMSE). The average RMSE values (see Table 7) were relatively low, indicating high consistency and accurate predictions (Leong et al., 2013; Raut et al., 2018).

**Table 7:** RMSE values for training and testing routines.

Neural network	Model A		Model B		Model C	
	Input: CQ, ITF, TTF		Input: CQ, ITF, TTF		Input: CQ, ITF, TTF, PA, PC, LS, SS	
	Output: PA		Output: PC		Output: SE	
	Training	Testing	Training	Testing	Training	Testing
ANN1	0.330	0.258	0.323	0.345	0.221	0.140
ANN2	0.333	0.304	0.333	0.472	0.188	0.132
ANN3	0.339	0.258	0.346	0.331	0.169	0.131
ANN4	0.334	0.231	0.354	0.267	0.168	0.095
ANN5	0.333	0.419	0.334	0.283	0.196	0.160
ANN6	0.314	0.375	0.348	0.198	0.162	0.174
ANN7	0.320	0.417	0.333	0.264	0.207	0.132
ANN8	0.328	0.131	0.313	0.139	0.217	0.144
ANN9	0.330	0.302	0.351	0.200	0.169	0.140
ANN10	0.303	0.261	0.320	0.188	0.193	0.126
Mean	0.326	0.296	0.336	0.269	0.189	0.137
SD	0.104	0.299	0.118	0.311	0.147	0.145

The sensitivity analysis, as shown in Table 8, assesses the relative importance of independent variables in influencing the ANN model’s predicted values. Normalized importance was calculated to compare each factor’s contribution relative to the most significant predictor. In the PA model, TTF (normalized importance = 100%) emerged as the strongest predictor, followed by CQ (88.3%). In the PC model, TTF again showed the highest influence (100%), with CQ (74.6%) also playing a key role. For the SE model, the most influential variables were TTF (100%), SS (77.3%), and CQ (71.8%), highlighting the importance of both technological fit and school support in driving student engagement.

**Table 8:** Sensitivity analysis.

Neural network	Model A (Output: PA)			Model B (Output: PC)			Model C (Output: SE)						
	CQ	ITF	TTF	CQ	ITF	TTF	CQ	ITF	TTF	PA	PC	LS	SS
ANN1	0.288	0.058	0.654	0.535	0.116	0.349	0.306	0.058	0.245	0.102	0.100	0.124	0.065
ANN2	0.443	0.047	0.510	0.331	0.070	0.600	0.123	0.035	0.222	0.117	0.237	0.027	0.240
ANN3	0.486	0.187	0.327	0.090	0.439	0.471	0.224	0.072	0.282	0.072	0.104	0.116	0.131
ANN4	0.526	0.034	0.440	0.306	0.146	0.547	0.173	0.042	0.207	0.106	0.139	0.118	0.215
ANN5	0.412	0.051	0.537	0.368	0.346	0.286	0.163	0.050	0.191	0.133	0.101	0.047	0.315
ANN6	0.43	0.156	0.414	0.376	0.042	0.582	0.172	0.073	0.181	0.110	0.170	0.138	0.157
ANN7	0.548	0.092	0.360	0.356	0.227	0.416	0.118	0.108	0.217	0.136	0.137	0.117	0.167
ANN8	0.383	0.009	0.608	0.332	0.293	0.376	0.092	0.200	0.342	0.060	0.190	0.049	0.066
ANN9	0.332	0.077	0.591	0.276	0.220	0.504	0.134	0.085	0.174	0.135	0.196	0.095	0.181
ANN10	0.394	0.243	0.363	0.350	0.329	0.322	0.144	0.059	0.236	0.085	0.173	0.064	0.239
Relative importance	0.424	0.095	0.480	0.332	0.223	0.445	0.165	0.078	0.230	0.106	0.155	0.090	0.178
Normalized importance (%)	88.300	19.859	100	74.557	50.034	100	71.789	34.044	100	45.973	67.349	38.964	77.318

### 4.5 Configurations analysis: fsQCA results

To complement the linear and nonlinear analyses, fsQCA was applied to identify multiple pathways that lead to high student engagement. This method accounts for causal complexity and asymmetry, allowing for the identification of equifinal configurations that can each achieve the outcome of interest (Ragin, 2009). Prior to fsQCA, all variables were calibrated into fuzzy sets using the direct method proposed by Moreno et al. (2016). For each construct, we defined three qualitative anchors to determine full membership (0.95), crossover point (0.5), and full non-membership (0.05). The analysis yielded five configurations (see Table 9) that explain high levels of engagement with AI teaching assistants in design theory education. Each configuration represents a unique and sufficient path to high engagement, suggesting that different groups of students may benefit from different combinations of factors.

**Table 9:** fsQCA results.

Config.	Solution				
	1	2	3	4	5
CQ	●	●		●	⊗
ITF			⊗	●	●
TTF	●	●	●	●	●
PA	⊗	●	●	●	⊗
PC	⊗	●	●		●
LS	●	⊗	●	●	●
SS	●		●	●	●
Consistency	0.954	0.961	0.977	0.986	0.979
raw coverage	0.263	0.283	0.307	0.487	0.237
unique coverage	0.029	0.05	0.025	0.182	0.007
Overall solution coverage	0.639				
Overall solution consistency	0.963				

Black circle (●) indicates the presence of a condition; crossed-out circle (⊗) indicates the absence/negation whereas blank space denotes “do not care” condition.

To assess the quality of the model, consistency and coverage scores were used. All configurations and the overall solution exceed the recommended thresholds for consistency (>0.80) and coverage (>0.20), indicating that the model is reliable and explains a substantial proportion of the outcome (Rasoolimanesh et al., 2021). These results demonstrate that AI teaching assistants can enhance engagement through various mechanisms. The configurations will be discussed in detail in the discussion section.

### 4.6 Thematic analysis of interview data

To complement the quantitative findings, a thematic analysis was conducted on qualitative interview data collected from students who interacted with the AI teaching assistant in design theory courses. We identified three major themes:

#### 4.6.1 Theme 1: accessibility and efficiency as practical advantages

Students frequently praised the AI assistant’s immediate availability and fast response time. The AI teaching assistant was perceived as especially helpful during self-study sessions, homework preparation, and late-night revisions.

*“The AI assistant is always online. Whether I’m in the library, the studio, or my dorm, I can ask questions and get instant answers.” (P2) “Its biggest strength is the speed and breadth of information, especially when I’m working on short essays and need to quickly look up concepts.” (P4)*

This flexibility fits well within the self-directed, iterative nature of design theory learning, where students often need to revisit historical references or compare design movements on demand.

#### 4.6.2 Theme 2: gaps in depth, interpretation, and critical thinking support

While students appreciated the AI teaching assistant for basic explanations and surface-level summaries, many expressed dissatisfaction with its limited ability to generate nuanced interpretations, provoke critical reflection, or support the development of design thinking skills.

*“The AI gives generic answers, but when it comes to design thinking tasks that require multiple perspectives, it often misses the point. For example, when I asked about the controversies around a particular design movement, it just listed features without helping me reflect on why these debates exist.” (P5)*

These concerns suggest that while the AI teaching assistant is effective in transmitting knowledge, it lacks the dialogical and conceptual depth needed for more advanced engagement, such as analyzing design theories, contextualizing historical styles, or discussing aesthetics.

#### 4.6.3 Theme 3: critical trust and verification behavior

Many students reported being cautious about the AI teaching assistant’s accuracy and described strategies for cross-checking information using textbooks, the lecturer’s slides, or other AI tools. Rather than fully trusting the AI teaching assistant, they saw it as a helpful but fallible supplement.

*“Sometimes its answers aren’t quite right, so I check my textbook, change other AI tools or ask the teacher. You can’t blindly trust it.”(P1)*

This theme illustrates a reflective use of AI, where learners actively verify content. It aligns with design education values such as self-direction, critique, and iterative inquiry. Importantly, the AI teaching assistant was not viewed as a replacement for teachers, but rather a complement that required critical engagement.

## 5 Discussion of findings

**Figure 6:**
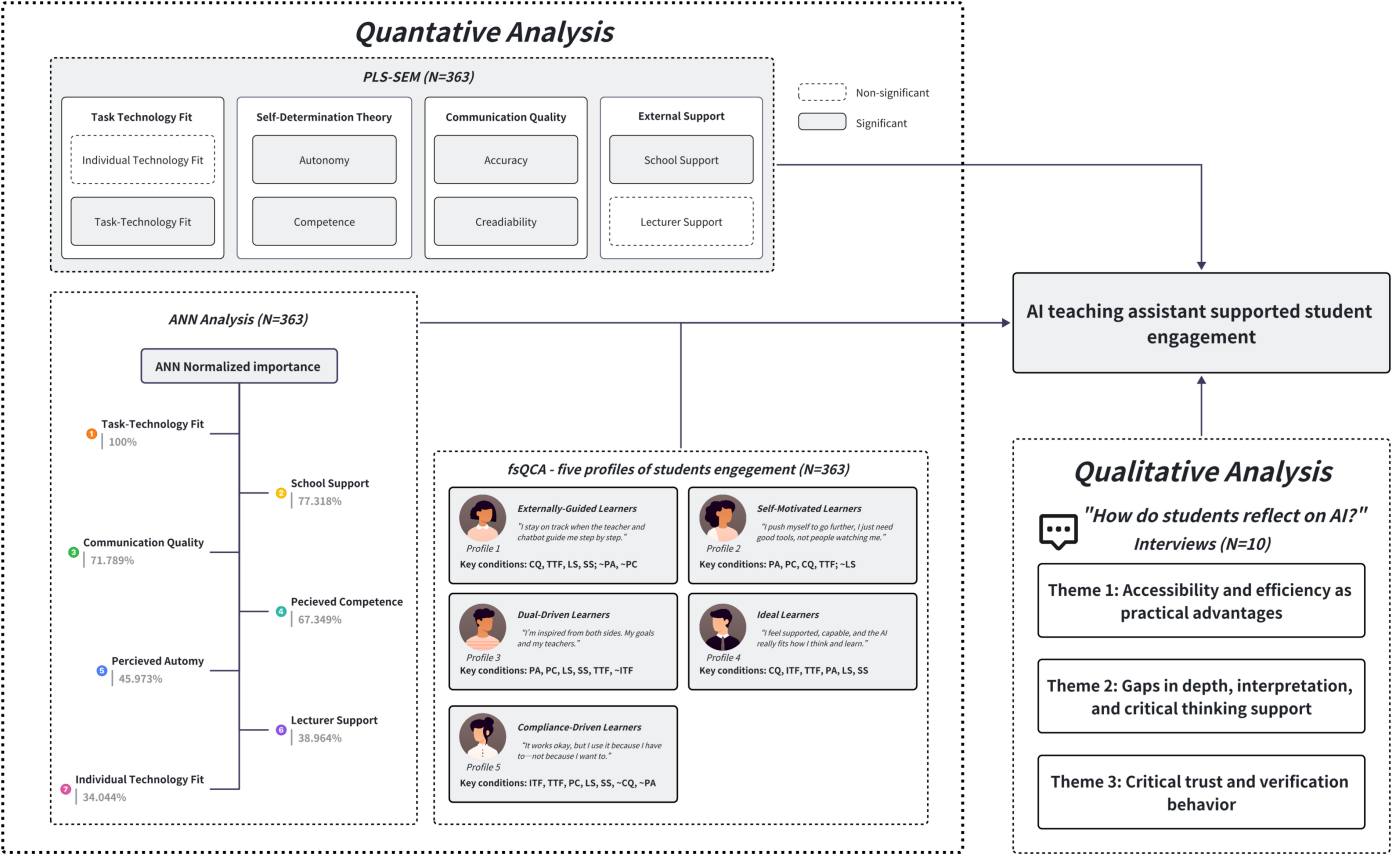
A mixed-methods model of AI-supported student engagement in design education.

To provide a holistic view of the study’s key findings, Figure 6 presents an integrated model that synthesizes the results from quantitative analyses (PLS-SEM and ANN), configurational analysis (fsQCA), and qualitative thematic insights. This diagram serves as a visual scaffold for the discussion that follows, highlighting the multifaceted pathways and learner profiles contributing to student engagement with AI teaching assistants in design education.

### 5.1 Factors influencing student engagement with AI teaching assistants (RQ1–RQ4)

The results from the PLS-SEM, ANN, and fsQCA analyses revealed that student engagement with AI teaching assistants in design theory education is shaped by a combination of psychological, technological, and contextual factors. Among these, perceived competence (PC), perceived autonomy (PA), communication quality (CQ), task-technology fit (TTF), and school support (SS) emerged as the most significant predictors.

Our psychological constructs drawn from Self-Determination Theory (SDT) showed strong explanatory power. Both PA (*β* = 0.127, *p* < 0.05) and PC (*β* = 0.196, *p* < 0.001) had significant direct effects on student engagement, aligning with SDT’s emphasis on intrinsic motivation through autonomy and competence satisfaction (Chiu et al., 2024; Ryan and Deci, 2020). These findings are in line with studies in digital education contexts, showing that autonomy-supportive environments foster deeper engagement and self-regulation (Fryer et al., 2019). In design education, where student-led exploration and iterative learning are central (Dorst, 2011), the ability to independently use the AI assistant to seek clarification or explore ideas supports these core pedagogical goals. ANN results reinforced these findings: perceived competence (PC) and perceived autonomy (PA) were among the most influential predictors of engagement, with PC (67.349%) and PA (45.973%). Furthermore, fsQCA analysis showed that PA and/or PC were present in three out of five engagement configurations, indicating their crucial role across diverse learner profiles. These results align with prior studies in digital education showing that autonomy-supportive environments foster deeper engagement and self-regulation.

In our technological constructs, the communication quality of the AI assistant had a substantial effect on student engagement (*β* = 0.177, *p* < 0.01) and strongly influenced SDT constructs (PA and PC). ANN sensitivity analysis further highlighted CQ as one of the top predictors (71.789%). In the fsQCA configurations, CQ appeared as a core condition in three out of five pathways (Solutions 1, 2, 4), reinforcing that students’ perception of the AI teaching assistant’s responsiveness, accuracy, and credibility is critical for their engagement. In line with previous research, this suggests that students’ perception of the AI teaching assistant as a competent, credible, and responsive communicator plays a critical role in facilitating their use of the tool (Chiu et al., 2024), supporting prior research on the significance of interaction quality in digital learning (Saifi et al., 2025). In design theory education, where abstract concepts and contextual knowledge require AI teaching assistants to have clear articulation, communication quality becomes even more critical to help students grasp difficult content.

Third, TTF showed a strong and significant influence on engagement (*β* = 0.207, *p* < 0.001) and was ranked highly in the ANN sensitivity analysis (100% normalized importance across models predicting PA, PC, and SE). Critically, TTF was the only condition present in all five fsQCA configurations, indicating that students are most engaged when they perceive the AI assistant as well-aligned with their learning tasks. This is consistent with prior studies suggesting that students are more engaged when tools are well integrated with learning activities (Al-Emran et al., 2025; Dahri et al., 2024). In the context of design theory, where students are often involved in timelines, terminology, and theory, a well-matched AI assistant should streamline their workflow, providing quick access to examples or definitions that support rather than disrupt their conceptual reasoning.

Interestingly, individual technology fit (ITF) did not significantly predict student engagement in the PLS-SEM model (*β* = 0.054, *p* > 0.05). This suggests that being comfortable with digital technologies does not automatically translate into active engagement with an AI teaching assistant in design theory courses. Many design students are already accustomed to using complex digital tools, such as Adobe Illustrator or Photoshop for graphic design or Rhino and Blender for 3D modeling and rendering. As a result, their general digital literacy is relatively high, but this does not guarantee that they will find value in using a text-based AI assistant. What they seem to care about more is whether the tool truly supports their learning goals. For example, helping them analyze key design movements, understand theoretical frameworks, or apply abstract principles to design critiques.

Contextual factors also played a key role. School support (SS) had a significant direct effect on engagement (*β* = 0.216, *p* < 0.01) and emerged as an important factor in ANN (77.318% normalized importance). In fsQCA, SS was present in four out of five configurations (Solutions 1, 3, 4, 5), underscoring its broad impact across different learner types. These results suggest that institutional endorsement and integration of AI tools can encourage student buy-in. This is aligned with previous research (Molefi et al., 2024), presenting that school support is crucial to drive students to use AI tools. However, Lecturer Support (LS) did not reach statistical significance, which may reflect the self-driven nature of design disciplines, where students often engage in “learning by doing.” Notably, while lecturer support did not show a significant effect in the PLS-SEM model, it appeared in several configurations identified through fsQCA. This apparent discrepancy reflects the different logics of the two methods: while SEM captures linear, net effects (Hair et al., 2019), fsQCA identifies condition combinations (Ragin, 2009) that are sufficient for high engagement. Lecturer support, though not impactful on its own, can act as a complementary facilitator when paired with strong school support, task-technology fit, or student competence. In theory-based design courses, learners tend to take ownership of concept exploration, particularly when assignments demand individual interpretation or application. In contrast, visible institutional support, such as technical training, resource access, or official endorsement, plays a more important role in legitimizing and encouraging the use of AI teaching assistants (Al-Emran et al., 2025).

Collectively, these results highlight that while individual motivational states and perceptions of technology quality are important, broader institutional integration and task alignment are equally vital. Multi-method triangulation (PLS-SEM, ANN, fsQCA) strengthens confidence in these findings by demonstrating convergence across statistical modeling, machine learning, and configurational analysis. Table 10 summarizes the cross-validation outcomes, revealing consistent predictors and demonstrating the complementary nature of psychological, technological, and contextual factors in shaping student engagement with AI teaching assistants.

**Table 10:** Integrated triangulation results: psychological, technological, and contextual predictors of student engagement.

Construct	PLS-SEM (Direct effect)	ANN (Relative importance)	fsQCA (Presence in configurations)	Interpretation summary
Perceived Autonomy (PA)	Significant (*β* = 0.127, *p* < 0.05)	45.973% (Moderate predictor)	Present in 3 of 5 configurations (Solutions 2, 3, 4)	PA moderately predicts engagement individually, and is a necessary motivational condition in multiple configurational paths.
Perceived Competence (PC)	Significant (*β* = 0.196, *p* < 0.001)	67.349% (High predictor)	Present in 3 of 5 configurations (Solutions 2, 3, 5)	PC is a strong direct predictor and repeatedly appears as a core element facilitating engagement.
Communication Quality (CQ)	Significant (*β* = 0.177, *p* < 0.01)	71.789% (High predictor)	Present in 3 of 5 configurations (Solutions 1, 2, 4)	CQ plays a substantial role in direct influence and consistently enhances engagement when present with other factors.
Task-Technology Fit (TTF)	Significant (*β* = 0.207, *p* < 0.001)	100% (Most influential predictor)	Present in all 5 configurations (Solutions 1–5)	TTF is the most robust factor, showing dominant linear, nonlinear, and configurational effects across all engaged profiles.
Individual Technology Fit (ITF)	Not Significant (*β* = 0.054, *p* > 0.05)	34.044% (Moderate-low predictor)	Present in 2 of 5 configurations (Solutions 4, 5)	ITF has limited direct impact but acts as a supplementary contributor in specific learner pathways.
School Support (SS)	Significant (*β* = 0.216, *p* < 0.01)	77.318% (Very high predictor)	Present in 4 of 5 configurations (Solutions 1, 3, 4, 5)	SS strongly enhances engagement through environmental facilitation, validated by both SEM and fsQCA.
Lecturer Support (LS)	Not Significant (*β* = 0.073, *p* > 0.05)	38.964% (Moderate-low predictor)	Present in 4 of 5 configurations (Solutions 1, 3, 4, 5)	LS plays a complementary rather than primary role, boosting engagement in combination with other supports.

### 5.2 Configurational pathways and learner profiles for high engagement (RQ5)

While the general linear analysis above identifies individual effects, student engagement is a multifaceted outcome often arising from specific combinations of factors. We used fsQCA to capture this complexity to uncover multiple conjunctural paths leading to high engagement. This approach recognizes that there may be five distinct profiles of highly engaged learners, each with its own recipe of conditions. These configurations are summarized as follows:

#### 5.2.1 Profile 1: externally-guided learners (key conditions: CQ, TTF, LS, SS; ~PA, ~PC)

In this configuration, strong external support compensates for lower internal motivation. High levels of AI teaching assistant communication quality (CQ), lecturer support (LS), and school support (SS) were jointly present, while the students’ perceived autonomy and competence were low or absent. In other words, even if a student was not initially self-driven or confident, the combination of an excellent AI teaching assistant experience plus active encouragement from teachers and institutions encourages them to engage. We interpret this profile as students who engage because the environment strongly facilitates it.

#### 5.2.2 Profile 2: self-motivated learners (key conditions: PA, PC, CQ, TTF; ~LS)

This configuration was almost the mirror image of *Profile 1*. Students who followed this path had high intrinsic motivation—evidenced by high perceived autonomy (PA) and competence (PC)—along with a good task-technology fit (TTF) of the AI teaching assistant, but notably low lecturer support. Their engagement is motivated not by external structures but by a deep internalized interest in design theory content and their perceived capacity to master it. These were learners who engaged actively with the AI assistant largely on their own initiative, perhaps despite a less enthusiastic instructor. They found the AI teaching assistant fit their personal learning approach well, and their internal drive carried them. This profile underscores that highly motivated students will take advantage of the technology if it aligns with their needs, even without strong external prompting.

#### 5.2.3 Profile 3: dual-driven learners (key conditions: PA, PC, LS, SS, TTF, ~ITF)

In this ideal scenario, both psychological needs and external supports were present. High PA, high PC, and high support from both the lecturer and the school came together to yield consistently high engagement. Students fitting this profile had the full package: they were internally motivated and felt autonomous/competent, and they also benefited from encouragement and a well-supported platform. Engagement in this group was predictably strong and stable. We can view this as a “win-win” configuration, where institutional efforts and personal motivation amplify each other. A student in this category might say, *“I love design theory, and the MinArt AI gave me even more ways to learn. Our professor’s tips on using it and the university’s support made it seamless.”* Though not surprising, this profile confirms that maximizing both internal and external factors produces the best outcome.

#### 5.2.4 Profile 4: ideal learners (key conditions: CQ, ITF, TTF, PA, LS, SS)

This configuration included most measured conditions present. It represents an ideal-typical case where everything that could go right did go right. These students had an excellent experience on every front and thus, unsurprisingly, showed high engagement. Like *Profile 3*, the nuance here is the additional presence of communication quality and perfect task fit. It reflects a scenario where the technology, pedagogy, and student readiness are in complete harmony—an alignment that might be rare but is illuminating as a goal. This all-positive profile reinforces the notion that, to achieve optimal engagement, educators should strive for a holistic solution: a high-quality AI assistant embedded in a supportive learning ecosystem, used by students who are both motivated and trained to use it.

#### 5.2.5 Profile 5: compliance-driven learners (key conditions: ITF, TTF, PC, LS, SS, ~CQ, ~PA)

Interestingly, we identified a configuration where some core technological factors were absent (notably, low AI teaching assistant communication quality or low perceived autonomy in using it), yet engagement was high due to strong institutional support combined with student competence. In this profile, students did not rate the AI teaching assistant’s responses very highly (perhaps they found answers lacking depth or accuracy), and/or they did not feel the AI gave them much autonomy; however, they did feel confident in their own ability (high PC) and sensed that both the instructor and institution expected them to use the tool (high LS and SS). Essentially, these learners engaged with the AI assistant out of a sense of duty or external expectation and their own drive to succeed, rather than because they loved the tool itself. One way to interpret this is that for some students, even if the AI teaching assistant is not performing optimally, they will persist in using it if they are determined to do well in the course and know that using the tool is part of the course culture. This profile is somewhat cautionary, suggesting that students might tolerate suboptimal technology if other pressures or motivations are strong, but ideally, we do not want “engagement” to come at the expense of a poor experience.

These five profiles highlight the concept of equifinality in educational technology use (multiple different paths to the same outcome). Not all highly engaged students look the same; some are driven by their own enthusiasm, others by external support structures, and others by a mix of both. For educators and developers, this means there is no single formula to get every student engaged with an AI assistant. Different students may require different combinations of interventions. Therefore, embracing a configurational perspective allows instructors to recognize and foster multiple routes to engagement rather than expecting all students to respond to the AI intervention in the same way.

### 5.3 Student reflections on learning with AI teaching assistants (RQ6)

The qualitative interviews provided a nuanced view of how students experienced the AI teaching assistant in a design theory course. Three main themes emerged: (1) the AI teaching assistant’s immediacy and usefulness, (2) its limited capacity for deep inquiry, and (3) evolving student trust and critical engagement.

First, students appreciated the AI teaching assistant’s 24/7 availability, which allowed them to access support anytime during independent study. This aligns with previous research. It presents the accessibility of the AI teacher and the availability of support for students outside traditional classroom hours as one of the key benefits (Saihi et al., 2025). This enables students to seek help and clarification at their convenience (Roca et al., 2024). Many students liken it to having a personal tutor, especially helpful during late-night work. In the design process, this aligns with the “Discover” phase of the Double Diamond model, where learners seek information widely (Grönman and Lindfors, 2021). However, students also reflected that while the AI teaching assistant is good for quick answers, it often lacks the depth to support meaningful learning. This highlights the need to integrate the AI teaching assistant into learning designs that foster reflection and deeper engagement, which is especially important in design education, where understanding historical and theoretical context is key.

Second, while the AI teaching assistant performs well in knowledge delivery, its responses are often “flat” or lacking in critical depth. Students feel it rarely asks follow-up questions or challenges in their thinking unless prompted. This is in line with the views articulated in previous articles, where there is a consensus that AI-generated textual content appears logically weak, is not accurate enough in terms of facts and veracity, is not critical enough in terms of data exposition, and is not novel enough (Dwivedi et al., 2023). Instructors in design education often stimulate learning by asking, “Why do you think this works?” or “Have you considered this angle?,” questions that drive design thinking (Zhang et al., 2024). Although some students attempt to elicit deeper responses by framing open-ended questions, they still find the interaction less rich than classroom dialogue. In the future, one potential solution is to embed scaffolded prompt templates or critical thinking cues within the AI teaching assistant interface to guide students toward deeper inquiry. Instructors can also reinforce this by integrating AI teaching assistant responses into classroom critique and encouraging students to compare AI-generated responses with their own interpretations. Although the AI may not yet replace the nuanced dialogue of a human educator, it can become a valuable complement when situated within a reflective pedagogical structure. Nevertheless, this process of formulating better prompts became a reflective exercise in itself, helping students structure their thinking, similar to Schön’s concept of “reflection-in-action” in design studios (Schön, 1987).

Third, students develop stronger information literacy and critical habits over time. Initially, many students trust the AI teaching assistant fully, but gradually adopt a verification mindset, like cross-checking its answers against textbooks or other sources. Aligning with this, previous research criticizes that GenAI provides false information or exhibits phenomena of confabulation or hallucination (Arkoudas, 2023). However, in our research, we see a positive shift toward AI literacy—critical thinking, a key component of digital competence in today’s learning environments (Zhang and Zhang, 2024). Educators could enhance this by incorporating guide verification tasks or teaching strategies for assessing AI-generated content, essential practices in ethical and informed design.

Importantly, several students describe the AI teaching assistant as a “safe space” to ask questions they might avoid in class. This aligns with previous research (Pesonen, 2021; Roehrer et al., 2024). This anonymity supports self-confidence and reduces the fear of judgment, helping students engage more actively in learning. This also aligns with a method called “thinking out loud” in design education (Calder, 2018). The role of an AI teaching assistant as a non-judgmental thinking partner can be particularly beneficial in design education, which often requires emotional resilience and persistence through ambiguity.

However, concerns about over-reliance and ethical use also emerged. Some students worry that excessive use might limit their independent thinking. This is in line with prior research, suggesting that AI poses a risk to students in that their ability to think independently and express themselves verbally may decline (Dwivedi et al., 2023). Besides, other students question how their data was handled or express fears of unintentionally crossing academic boundaries (Cotton et al., 2024). These concerns align with broader calls in design education for responsible technology use and ethical engagement with tools, which need to be addressed in more specific future studies.

## 6 Theoretical and practical implications

This study offers important contributions to both theoretical development and practical application in the context of design theory education.

This study extends the application of Self-Determination Theory (SDT) and Task-Technology Fit (TTF) by constructing an integrated triadic model of motivation, technology, and contextual support in the context of design theory education. Unlike prior studies treating these factors separately (Bilquise et al., 2024; Yildiz Durak and Onan, 2024), we reveal how psychological needs (autonomy, competence), technological alignment, and institutional support jointly shape engagement. Notably, communication quality (CQ) emerges as a key technological factor bridging system attributes and motivational perceptions, highlighting its pivotal role in AI-assisted learning. Fourth, the study expands the application of AI education research into conceptually demanding and creativity-driven fields. While AI chatbots have been widely studied in procedural domains (e.g., language learning, STEM) (Ebadi and Amini, 2024; Nam and Bai, 2023; Rahimi et al., 2025), this study shows how they can be tailored to support higher-order cognitive processes like reflection, critical inquiry, and conceptual integration within design theory.

From a practical standpoint, the findings offer guidance for educators and institutions seeking to integrate AI assistants into design education. AI teaching assistants should not only deliver accurate information but also support key pedagogical goals such as critical thinking, independent exploration, and reflection. Institutions play a key role in normalizing AI use by offering access, training, and endorsement. Educators should shift from using AI as a passive information provider toward positioning it as a reflective conversational partner. Training students in critical questioning and validation strategies can transform AI teaching assistant interactions into opportunities for deeper learning rather than superficial answer-seeking.

## 7 Conclusion

This study examines how AI teaching assistants influence student engagement in design theory education using a mixed-methods approach. By combining Self-Determination Theory and Task-Technology Fit frameworks, the findings reveal that engagement is driven by a triad of psychological needs (autonomy, competence), technological alignment (task fit, communication quality), and institutional support.

The study contributes theoretically by uncovering multiple configurational pathways to engagement using fsQCA, demonstrating that different learner profiles achieve high engagement through varied combinations of motivation, technology, and support. Practically, it highlights that AI assistants can enhance self-directed learning and knowledge exploration in conceptual, inquiry-driven domains like design theory, but need thoughtful integration to stimulate deeper reasoning and reflective practice.

Overall, AI teaching assistants hold significant potential to enhance student engagement in design education, particularly when positioned not merely as information sources but as scaffolds that support autonomous inquiry and the integration of complex concepts. Future research should further investigate how AI can facilitate higher-order thinking and foster creative processes within design pedagogy.

## Data Availability

The original contributions presented in the study are included in the article/Supplementary material, further inquiries can be directed to the corresponding author.
