# Supplementary material for: Building a triadic model of technology, motivation, and engagement: a mixed-methods study of AI teaching assistants in design theory education
Source: Front Psychol. 2025 Jul 2;16:1624182. doi: 10.3389/fpsyg.2025.1624182 (PMC12265309; doi:10.3389/fpsyg.2025.1624182)
Supplement: Supplementary file 1 [file Data_Sheet_1.docx]

# **Appendix A**

# **Technical Documentation: Customization of AI teaching assistant**

This appendix provides technical documentation for the development, customization, and evaluation of the AI-powered teaching assistant “MinArt AI”, designed to support undergraduate students in design theory courses. The AI teaching assistant was developed using Baidu’s ERNIE Bot platform and fine-tuned with domain-specific instructional content. The documentation below includes details on knowledge base construction, model parameters, and evaluation methods, enabling future replication.

## **1. Base Model and Development Platform**

- Base Model: ERNIE Bot (文心一言), Baidu Large Language Model
- Platform Used: Baidu ERNIE Studio (API-based customization)
- Language: Chinese
- Access: <https://yiyan.baidu.com/>

## **2. Knowledge Base Construction**

To create a domain-specific AI teaching assistant for design theory education, a curated knowledge base was constructed using the following sources:

- Core textbooks: Excerpts from courses such as “History of Chinese and Foreign Design”,“Design Thinking”, and “Design Psychology”.
- Instructor lecture notes: Annotated PDF slide decks and transcripts from previous years.
- QA logs: Common student questions from past semesters collected via forums and feedback forms.
- External materials: Public resources such as Wikipedia articles, scholarly summaries.

**Data formats used**:

| **Content Type** | **File Format(s)** | **Notes** |
| --- | --- | --- |
| Lecture notes, textbooks | .docx, .pdf, .txt | Text files were preprocessed to remove formatting noise |
| Visual references (e.g., Red and Blue Chair) | .png, .jpg | Used in response enrichment with image descriptions |
| Case libraries / reference tables | .xlsx, .csv | Included theory tags, design principles, timelines |
| Voice materials (optional) | .mp3, .m4a | Converted to transcript-based FAQ for design history |

All files were uploaded to the “knowledge base” module of the Baidu platform. Text materials were pre-processed to remove excessive formatting and ensure clarity, and each document was tagged with relevant themes (e.g., Bauhaus, Gestalt principles) for optimal semantic matching.

## **3. Conversation Configuration**

The agent’s interaction behavior was set through the “Agent Settings” tab.

Key conversational configurations included:

1) Welcome Message, e.g.:

“Hello! I’m your design assistant, here to help you solve all kinds of design-related problems. Whether you’re working on product, interior, or visual design, I can offer advice and inspiration. Let me know what you need!”

2) Example Starter Questions (randomized display of 3 out of N options):

- “How do I design an eye-catching product package?”
- “What are the current trends in interior design?”
- “How can I draw inspiration from other designers?”

3) Auto-Generated Follow-up Prompts:

Enabled to help sustain engagement by suggesting 3 follow-up questions after each response.

4) Quick Action Buttons:

Displayed above the input box, e.g.:

- “View case study”
- “Search design styles”
- “Explain design concept”

5) Plugin Integration: Not enabled in the current version.

6) Search Function: Disabled to maintain knowledge base consistency.

## **4. Evaluation and Pilot Testing**

To evaluate the model’s readiness for deployment, we used the following procedure:

- Query Set: 100 representative design-related queries submitted by students
- Metrics: Accuracy, conceptual clarity, and contextual relevance
- Evaluation Team: Three faculty raters and 15 pilot students
- Feedback Integration: Iterative tuning was done after the first and second rounds of feedback

## **5. Deployment and Usage**

The finalized AI teaching assistant was deployed on Baidu's mobile learning portal and embedded into the course website.

- Deployment URL: <https://mbd.baidu.com/ma/s/0gjlz9Z7>
- Available 24/7 for student use via mobile or desktop browser

## **6. Replication Notes**

To replicate a similar AI teaching assistant in other domains:

1. To replicate this setup for other courses or subject areas:
2. Register at <https://agents.baidu.com>
3. Create a new “Intelligent Agent”.
4. Upload structured learning materials in formats like .docx, .pdf, .xlsx, .jpg, .mp3.
5. Set a clear and welcoming introductory message and define at least 3 starter questions.
6. Optionally enable automatic follow-up prompts and define quick-action buttons.
7. Publish the AI teaching assistant and share the public link with students or embed it in an LMS.
