# Supplementary material for: Building a triadic model of technology, motivation, and engagement: a mixed-methods study of AI teaching assistants in design theory education
Source: Front Psychol. 2025 Jul 2;16:1624182. doi: 10.3389/fpsyg.2025.1624182 (PMC12265309; doi:10.3389/fpsyg.2025.1624182)
Supplement: Supplementary file 2 [file Data_Sheet_2.docx]

# **Appendix B**

Table 11 Constructs and Items

| **Constructs** | **Code** | **Items** | **References** |
| --- | --- | --- | --- |
| Chatbot Communication Quality | | | |
| Accuracy | AC1 | Communication with the AI chatbot teaching assistant is very timely. | (Cheng & Jiang, 2021; Chung et al., 2020) |
|  | AC2 | Communication with the AI chatbot teaching assistant is very accurate. |  |
|  | AC3 | The information provided by the AI chatbot teaching assistant is very sufficient. |  |
|  | AC4 | The communication content of the AI chatbot teaching assistant is very complete. |  |
| Credibility | CRE1 | The AI chatbot teaching assistant is honest. | (Cheng & Jiang, 2021; Chung et al., 2020) |
|  | CRE2 | The AI chatbot teaching assistant is very trustworthy. |  |
|  | CRE3 | The AI chatbot teaching assistant is very ethical in interactions. |  |
|  | CRE4 | The behavior of the AI chatbot teaching assistant conforms to ethical and moral standards. |  |
| Task Technology Fit Theory | | | |
| Individual Technology Fit | ITF1 | I actively and independently use AI chatbot teaching assistants to complete learning tasks in design theory courses. | (Suhail et al., 2024; B. Wu & Chen, 2017) |
|  | ITF2 | I actively participate in course discussions and feedback activities through the AI chatbot teaching assistant. |  |
|  | ITF3 | I try to use the AI chatbot teaching assistant to help myself achieve excellent performance in the course. |  |
|  | ITF4 | I consciously use the AI chatbot teaching assistant for self-directed learning. |  |
| Technology Fit | TTF1 | The functions of the AI chatbot teaching assistant meet my learning needs in this course. | (Suhail et al., 2024; B. Wu & Chen, 2017) |
|  | TTF2 | Using the AI chatbot teaching assistant aligns with my learning style and habits. |  |
|  | TTF3 | I can easily understand how to use the various functions of the AI chatbot teaching assistant. |  |
|  | TTF4 | The AI chatbot teaching assistant is suitable for assisting me in completing learning tasks in design theory courses. |  |
|  | TTF5 | The AI chatbot teaching assistant can conveniently and quickly provide me with the course-related information I need. |  |
| Self-Determination Theory | | | |
| Perceived Autonomy | PA1 | I can express my own opinions on how to use the AI chatbot teaching assistant. | (T. K. F. Chiu, 2022; Standage et al., 2005) |
|  | PA2_R | I feel pressured when using the AI chatbot teaching assistant. |  |
|  | PA3 | I can freely express my thoughts on using the AI chatbot teaching assistant. |  |
|  | PA6 | I can basically use the AI chatbot teaching assistant according to my own ideas. |  |
|  | PA7_R | I have little opportunity for autonomous decision-making when using the AI chatbot teaching assistant. |  |
| Perceived Competence | PC1_R | When using the AI chatbot teaching assistant, I don't feel very competent. | (T. K. F. Chiu, 2022; Standage et al., 2005) |
|  | PC2 | Other classmates think I am good at using the AI chatbot teaching assistant to aid learning. |  |
|  | PC3 | By using the AI chatbot teaching assistant, I have learned some interesting new skills. |  |
|  | PC4 | In learning with the AI chatbot teaching assistant, I often feel a sense of achievement. |  |
|  | PC6_R | When using the AI chatbot teaching assistant, I often feel inadequate. |  |
| External Support | | | |
| Lecturer Support | LS1 | I use the AI chatbot teaching assistant to ask questions to my teacher. | (Gharrah & Aljaafreh, 2021) |
|  | LS2 | The AI chatbot teaching assistant allows me to communicate with teachers anytime, anywhere. |  |
|  | LS3 | I can complete and submit my learning tasks through the AI chatbot teaching assistant without going to school. |  |
|  | LS4 | The AI chatbot teaching assistant provides me with opportunities to communicate with teachers. |  |
|  | LS5 | Teachers encourage the use of AI chatbot teaching assistants for educational communication. |  |
| School Support | SS1 | My school is committed to using AI chatbot teaching assistants in teaching. | (Lai & Chen, 2011) |
|  | SS2 | My school supports my efforts to use AI chatbot teaching assistants in studying. |  |
|  | SS3 | The school strongly encourages the use of AI chatbot teaching assistants for teaching. |  |
|  | SS4 | My school will recognize my efforts in using AI chatbot teaching assistants. |  |
| Student Engagement | SE1 | When using the AI chatbot teaching assistant to learn this course, I feel energetic. | (Aljaloud et al., 2019; Hidayat-ur-Rehman, 2024; Hwang et al., 2013) |
|  | SE2 | I feel energized and capable when learning this course. |  |
|  | SE3 | I am always willing to participate in all learning activities for this course. |  |
|  | SE4 | I am enthusiastic about this course. |  |
|  | SE6 | I am proud of my learning in this course. |  |
|  | SE7 | I feel happy when I focus on learning this course. |  |
|  | SE8 | I am completely immersed in learning this course. |  |
